# Detection of Paroxysmal Atrial Fibrillation from Dynamic ECG Recordings Based on a Deep Learning Model

**DOI:** 10.3390/jpm13050820

**Published:** 2023-05-12

**Authors:** Yating Hu, Tengfei Feng, Miao Wang, Chengyu Liu, Hong Tang

**Affiliations:** 1 School of Biomedical Engineering, Dalian University of Technology, Dalian 116024, China; yatinghu@mail.dlut.edu.cn (Y.H.);; 2 Department of Psychiatry, Psychotherapy and Psychosomatics, Medical School, RWTH Aachen University, 52074 Aachen, Germany; 3 School of Instrument Science and Engineering, Southeast University, Nanjing 210096, China

**Keywords:** atrial fibrillation, electrocardiogram, deep learning, Transformer

## Abstract

Background and Objectives: Atrial fibrillation (AF) is one of the most common arrhythmias clinically. Aging tends to increase the risk of AF, which also increases the burden of other comorbidities, including coronary artery disease (CAD), and even heart failure (HF). The precise detection of AF is a challenge due to its intermittence and unpredictability. A method for the accurate detection of AF is still needed. Methods: A deep learning model was used to detect atrial fibrillation. Here, a distinction was not made between AF and atrial flutter (AFL), both of which manifest as a similar pattern on an electrocardiogram (ECG). This method not only discriminated AF from normal rhythm of the heart, but also detected its onset and offset. The proposed model involved residual blocks and a Transformer encoder. Results and Conclusions: The data used for training were obtained from the CPSC2021 Challenge, and were collected using dynamic ECG devices. Tests on four public datasets validated the availability of the proposed method. The best performance for AF rhythm testing attained an accuracy of 98.67%, a sensitivity of 87.69%, and a specificity of 98.56%. In onset and offset detection, it obtained a sensitivity of 95.90% and 87.70%, respectively. The algorithm with a low FPR of 0.46% was able to reduce troubling false alarms. The model had a great capability to discriminate AF from normal rhythm and to detect its onset and offset. Noise stress tests were conducted after mixing three types of noise. We visualized the model’s features using a heatmap and illustrated its interpretability. The model focused directly on the crucial ECG waveform where showed obvious characteristics of AF.

## 1. Introduction

Worldwide, atrial fibrillation (AF) is one of the most common arrhythmias clinically. AF is an arrhythmia with uncoordinated atrial electrical activation, and consequently, ineffective atrial contraction, with electrocardiogram (ECG) characteristics of (1) irregular R-R intervals (when atrioventricular conduction is present), (2) an absence of distinct repeating P waves, and (3) irregular atrial activity [1]. A global total of 43.6 million individuals had prevalent AF/AFL in 2016. The currently estimated prevalence of AF in adults is between 2% and 4% [2], and a 2.3-fold rise [3] in AF prevalence is expected [4,5]. Aging tends to increase the risk of AF, which also increases the burden of other comorbidities, including coronary artery disease (CAD), and even heart failure (HF).

The recommendation for the diagnosis of AF is a standard 12-lead ECG recording or a single-lead ECG tracing of more than 30 s of heart rhythm; if AF is present, P waves will be absent and there will be an observable irregularity of R-R intervals [6]. AF is commonly classified into paroxysmal AF, persistent AF, long-standing persistent AF, and permanent AF. Clinically, asymptomatic AF patients occupy a certain proportion of patients, and need extra examinations for their diagnosis to be confirmed by physicians. In addition, the sudden onset and offset of AF also reduce the possibility of prediction. In this case, more time is spent on diagnosis, and patients take more risks. Although AF is not lethal, its effective management can prevent thromboembolism (including stroke) and tachycardia-mediated cardiomyopathy, control ventricular rate, etc.

The precise detection of AF is a challenge due to its intermittence and unpredictability. Resting ECG has been proven to be insufficient for detecting AF, since it has quite a short duration compared with dynamic ECG. Previously, physicians diagnosed AF after checking ECG records collected using a Holter monitor, and the inspections were time-consuming and repetitive. In recent years, we have witnessed the development of machine learning and deep learning (DL). With the aid of both algorithms and hardware resources, physicians can concentrate on performing diagnoses, and patients do not have to wait such a long time for the determined diagnosis.

The detection of AF has advanced significantly over the past decade, bringing it closer to practical application. Traditional approaches focused on unpresented P waves and irregular R-R intervals. Previous research has proposed multiple features to describe the above characteristics of ECG waveforms from time domain, the frequency domain, and even the joint time–frequency domain [7,8,9]. Refs. [10,11,12] have proposed multiple methods to delineate P wave characteristics in AF detection. The precise recognition of P waves is based on the premise that the ECG is clean and uncontaminated, which is almost impossible when using dynamic and wearable ECG collection in the real-life scenarios. A weak P wave becomes silent when affected by motion artifacts and myoelectric interference. Moreover, R wave detection for dynamic ECG recordings is not as accurate as that for resting ECG, which has a further negative effect on feature extraction, whereas algorithms based on deep learning translate the data into a more distinguishable pattern instead of extracting and sifting features. Deep networks have the intelligence to skip less important parts of the data and pay attention to the parts that correlate more closely with what we are interested in. A convolutional neural network (CNN) automatically extracts hierarchical features from the input data, which has been proven remarkable in computer vision. Xia et al. [13] were the first to apply a CNN to the detection of AF, and its performance was not dependent on P or R waves, even with hand-crafted features. A recurrent neural network (RNN) has the advantage of handling temporal problems, and hierarchical attention mechanisms interpreted the AF detection results with multiple levels of resolution in Ref. [14]. The bidirectional long short memory (LSTM) designed by Faust et al. [15] was the first to detect AF based on heart rate signals. It effectively learned and extracted features from R-R interval input data composed of 100-beat segments, and attained accuracy levels of 98.51% and 99.77%, respectively. Cai et al. [16] constructed a one-dimensional deep densely connected neural network (DDNN) to detect AF with a length of 10 s, and obtained an accuracy of 99.35 ± 0.26%, a sensitivity of 99.19 ± 0.31%, and a specificity of 99.44 ± 0.17%. Research has also been carried out to detect AF in a single cardiac cycle. Fan et al. [17] combined the heart’s rhythmic information and morphological features, captured using U-Net and a 34-layer residual network, respectively. An F1 value of 85.08 ± 0.99% and an accuracy of 87.22 ± 0.71% were achieved. Baalman et al. [18] developed a morphology-based DL model to discriminate AF from sinus rhythm (SR), and to visualize which parts of the ECG were used by the model to derive the right classification. Most previous papers made progress in the detection of AF rhythm, but the locations where AF started and ended were not given enough attention. AF burden is closely dependent on AF onset and offset, since it is computed using AF duration and the density of episodes per unit of time. The severity of AF burden reflects the development of chronic arrhythmia.

The aim of our study is to propose a novel AF detection method for a short-term ECG sample, which means we intend to predict not only the types of heartbeat, but also the location of AF in long-term ECG recordings. The deep learning model, based on residual blocks and a Transformer encoder, showed good performance on several public databases. The paper is organized as follows. Section 2 introduces the databases used for the experiment and details of the proposed method. In Section 3 and Section 4, we provide a performance evaluation of the deep learning model and discuss the given results. Finally, we present our conclusions and declared the study’s limitations.

## 2. Materials and Methods

### 2.1. Databases

Five different databases were used for training and testing, including the CPSC2021 dataset (CPSC2021) [19], the MIT-BIH Arrhythmia Database (MITDB) [20], the Long-Term AF Database (LTAF) [21], the MIT-BIH Atrial Fibrillation Database (AFDB) [22], and the CinC2017 dataset [23].

For CPSC2021, the data are recorded using 12-lead Holter or 3-lead wearable ECG monitoring devices, which are variable-length ECG recording fragments extracted from lead I and lead II of a long-term dynamic ECG. The sample frequency was 200 Hz. The onsets and offsets of AF are given in WFDB format. The whole dataset consists of 1436 records, which can be classified into three rhythm types: non-AF rhythm, persistent AF rhythm, and paroxysmal AF rhythm. A total of 732 records are normal, while 475 records are persistent AF, and 229 records are paroxysmal AF. Paroxysmal AF records present several episodes with various durations, whereas persistent AF shows obvious AF characteristics from the first cardiac cycle to the last.

MITDB is one of the most widely used databases in the automatic detection of rhythm. It contains 48 half-hour excerpts of two-channel ambulatory ECG recordings, obtained from 47 subjects studied by the BIH Arrhythmia Laboratory between 1975 and 1979. The recordings are digitized at 360 Hz per channel with 11-bit resolution over a 10 mV range [20]. The subjects are chosen from inpatients and outpatients, and some of them have less common but clinically significant arrhythmias.

LTAF includes 84 long-term ECG recordings of subjects with paroxysmal or sustained AF. Each record contains two simultaneously recorded ECG signals digitized at 128 Hz with 12-bit resolution over a 20-mV range; record durations vary but are typically 24 to 25 h [21].

AFDB was published in 2000 and includes 25 long-term ECG recordings of human subjects with AF, most of which are paroxysmal AF. The individual recordings are 10 h in duration, and contain two ECG signals that are sampled at 250 Hz with a 12-bit resolution over a range of ±10 millivolts [22]. The involved rhythms are AFIB (atrial fibrillation), AFL (atrial flutter), J (AV junctional rhythm), and N (all other rhythms).

The 2017 PhysioNet/CinC Challenge provided a single-lead ECG dataset that includes normal sinus rhythm, AF, other alternative rhythms, and noisy segments. The ECG recordings, collected using an AliveCor device, contain 8528 single-lead ECG recordings lasting from 9 s to just over 60 s. The recordings are sampled at 300 Hz and they have been band-pass filtered using the AliveCor device [23]. The statistics of the used databases are shown in Table 1.

### 2.2. Data Preprocessing

ECG recordings were first preprocessed through filters to eliminate the baseline wander and high-frequency noises. Baseline wander was filtered through median filters with window sizes of 0.2 s and 0.6 s. A Butterworth low-pass filter with a cut-off frequency of 40 Hz kept the main frequency band of ECG signals. The impulse noise was also taken into consideration and was totally removed by eliminating the exception value.

**Figure 1:**
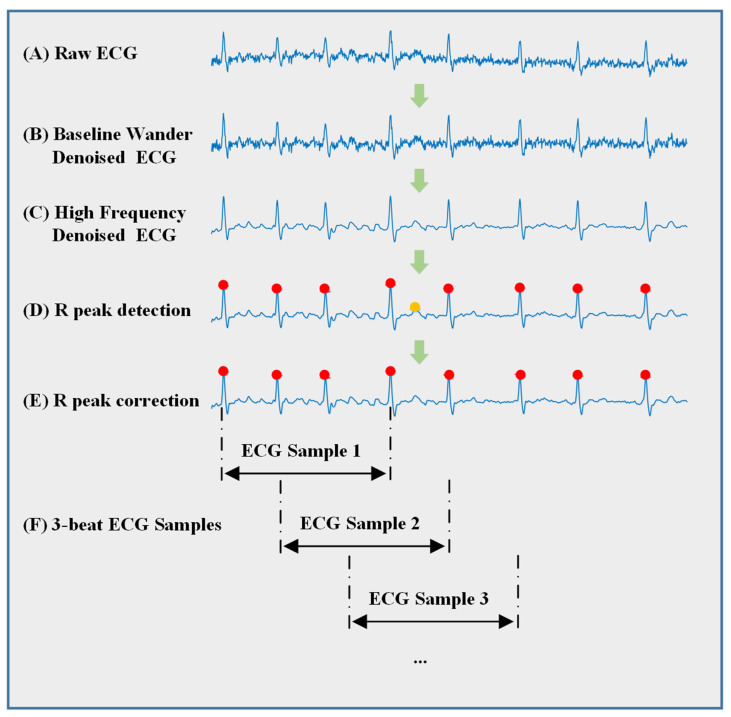
Preprocessing of a persistent ECG recording. (**A**) Raw ECG from CPSC2021 dataset. (**B**) Baseline wander-denoised ECG after clearing the baseline wander. (**C**) High-frequency-denoised ECG after clearing the high frequency noise. (**D**) R peak detection. (**E**) R peak correction. In this step, the correction mechanism was used to improve the precision. (**F**) The last step, where we split the recording into ECG samples. Every sample is stretched or squeezed to 600 data points instead of using padding to magnify the waveform characteristics.

The Pan–Tompkins algorithm with a correction mechanism was used to locate R peaks. R-R intervals become irregular when AF occurs, which causes some cardiac cycles to shorten. In addition, there exists misrecognition leading to particularly short R-R intervals in dynamic ECG detection. As a result, we searched for ECG recordings with a maximum R-R interval of longer than 0.5 s, and removed incorrect R-peaks, which obviously had lower amplitudes compared with the prior and the latter peaks. ECG reveals the electrophysiological characteristics of heart, especially heart rate variability (HRV). Each heartbeat has a distinct time duration, which leads to ECG data being out of alignment. However, the input of the network must be organized, so we performed a time-scaling strategy to obtain ECG samples of the same length. Padding is frequently employed to create structured data for networks; however, in order to emphasize the ECG waveform difference between R peaks, we stretched or squeezed the ECG samples. Every ECG sample included three adjacent cardiac cycles and was linearly scaled to 600 data points in the time domain instead of using padding. On average, there were approximately 200 data points in each heartbeat, which was enough to identify the main characteristics of the ECG waveform. We split ECG samples according to R peaks, so the definition of onset is the R peak of the nearest heartbeat before AF occurs, and offset is the R peak of the last heartbeat after AF terminates. We classified 3-beat ECG samples into two categories. If one of the three beats in a sample was AF, then the category of this sample was labeled as AF. Otherwise, it was labeled as non-AF. Figure 1 illustrates the preprocessing of a persistent AF ECG recording. We first eliminated baseline wander, and then, removed high-frequency noise and identified the R-peaks. Finally, we split the recording into ECG samples, which were all labeled as AF.

### 2.3. Methods

**Figure 2:**
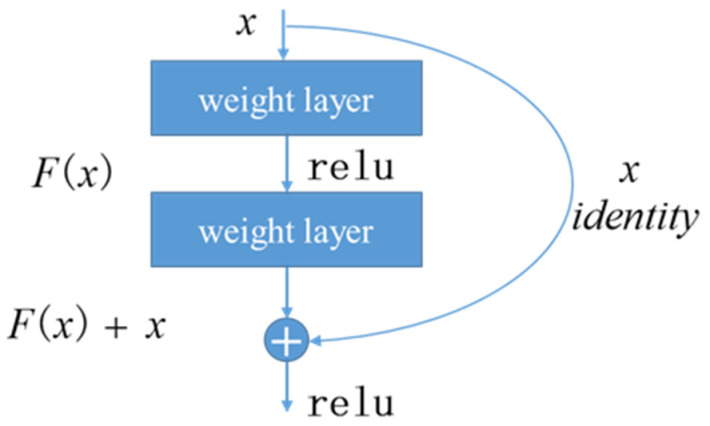
Structure of residual block [24]. Vector *x* is an input of the residual block.

The proposed deep learning model was built based on residual blocks and a Transformer encoder. Residual blocks, proposed by He et al. [24] in 2015, addressed the degradation problem by introducing a deep residual learning framework. As the depth of neural network increased, the training process became more and more difficult. As a result of the increasing loss during training, the deeper network did not show the expected performance. Residual blocks with shortcuts enabled the deep neural network with up to a hundred layers easier to optimize; consequently, competitive accuracy was obtained. The structure of a residual block is shown in Figure 2. Vector *x* was an input of the residual block. Here, identity mapping was set as the shortcut, so the output of the block should be the sum of *x* and the output of two weight layers. Shortcut connection skipped the weight layers and performed identity mapping, avoiding the vanishing gradient problems.

**Figure 3:**
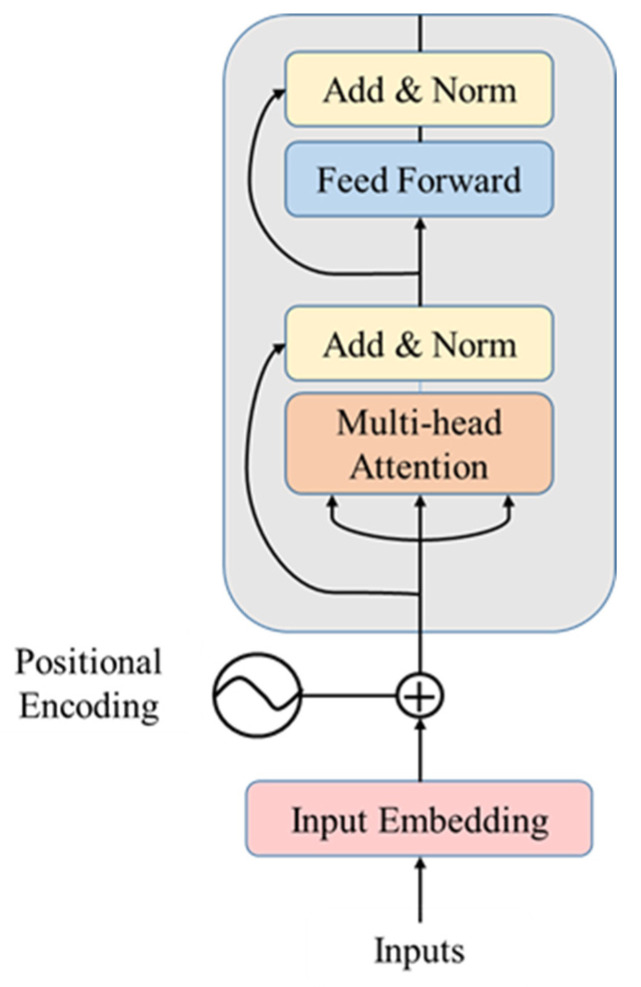
Model Structure of the Transformer encoder [25]. The input embedding with position encoding flows into N encoders, passing through the multi-head self-attention layer and feed-forward network.

Vaswani et al. [25] proposed the Transformer, a model architecture eschewing recurrence, and instead, relying entirely on an attention mechanism to draw global dependencies between the input and output. It has been proven to have excellent performance in machine translation [26] and computer vision, such as image recognition [27,28], object detection [29,30], and segmentation [31]. The model structure of the Transformer encoder is shown in Figure 3. In this paper, we only used the encoder part, consisting of two sub-layers: a multi-head self-attention mechanism and a position-wise fully connected feed-forward network. Residual connections were employed around these two sub-layers. Each of the sub-layers then underwent layer normalization.

**Figure 4:**
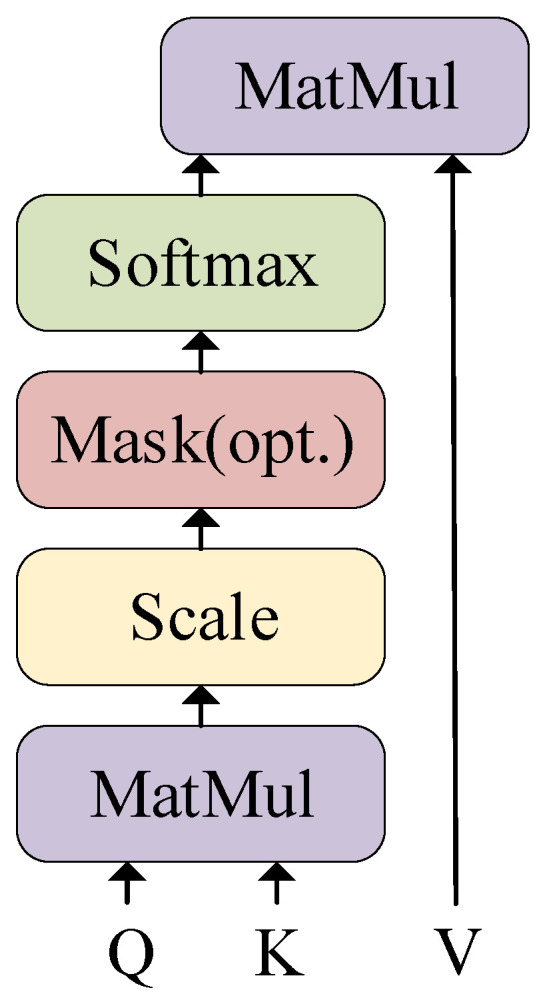
Scaled dot-product attention [25].

An attention mechanism was used to map the query and the pairs of key values into the output. According to the similarity of query and key, the weights of key values were computed, and then, the output could be calculated as a weighted sum of the values. The traditional attention mechanism can be represented as:

(1) attention_output=Attention(Q,K,V)

where *Q*, *K*, and *V* represent a query, key, and value, respectively. Self-attention, sometimes called intra-attention, is an attention mechanism that relates different positions of a single sequence in order to compute a representation of the sequence. Therefore, multi-head self-attention further allows the model to jointly attend to information from different representation subspaces at different positions due to the increasing dimension of the head. Ref. [25] used a particular attention mechanism called “Scaled Dot-Product Attention” (Figure 4). The dimensions of keys and values were defined as *d_k_* and *d_v_*. The Transformer considered *d_k_* as a scaling factor, and the matrix of the output was computed as:

(2) $$\mathrm{Attention}(Q,K,V)=\mathrm{softmax}(\frac{QK^{T}}{\sqrt{d_{k}}})V$$

**Figure 5:**
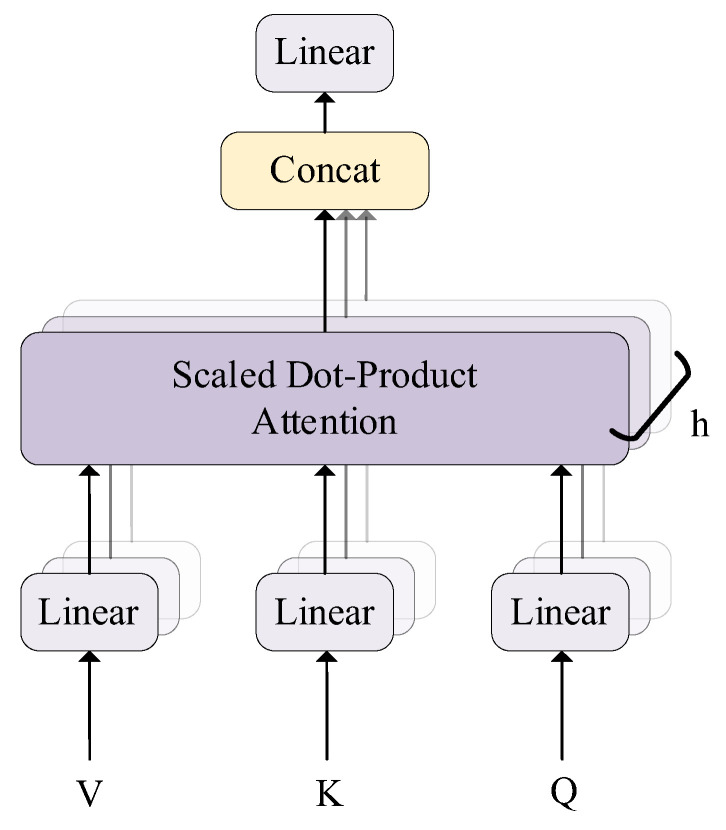
Multi-head self-attention [25].

Firstly, the dot products of the queries with all keys were computed, and then, divided by a scaling factor *d_k_*. A SoftMax function was employed to obtain the final weights. On account of simultaneous computation, queries, keys, and values were all packed into the matrices *Q*, *K*, and *V*, which largely reduced the time cost when compared with additive attention. The scaling factor *d_k_* solved the small gradient problem when the dot products became extremely large. Though attention mechanisms were used, it may not be possible to completely describe all the dependencies using only one attention function [32]. It was advantageous to linearly project the queries, keys, and values *h* times for more latent dependencies. As shown in Figure 5, the matrices *Q*, *K* and *V* were projected using different and learned linear projections. Each different projection of queries, keys, and values was followed by a scaled dot-product attention mechanism. The results of the attention functions were concatenated, and finally, projected again. Multi-head self-attention was represented as:

(3) $$\begin{array}{l} \mathrm{MultiHead}(Q,K,V)=\mathrm{Concat}({\mathrm{head}}_{1},\ldots {,\mathrm{head}}_{h})W^{O}\\ {\mathrm{where}\;\mathrm{head}}_{i}^{}=\mathrm{Attention}(QW_{i}^{Q},KW_{i}^{K},VW_{i}^{V}) \end{array}$$

The position-wise feed-forward network was composed of two linear transformations and a ReLU activation function. In addition, position encoding was added to compensate for the absence of position information in sequences. Sine and cosine functions were chosen because they made the learning of relative positions easier using a linear relation within any fixed offset [33].

In brief, a Transformer focuses on the global view of the input, and has the strength to model distant independence between data points. These merits support the application of the Transformer to physiological signals, especially in the detection of AF. When AF occurs, R-R intervals become irregular. A very short heartbeat often follows a long heartbeat, and vice versa. This phenomenon is fairly common if AF suddenly starts or terminates. The advantage of the Transformer lies in its obvious capability for griping the strong dependencies of several heartbeats. Unlike RNNs, in which temporal dependencies are embedded in the intermediate features and carried from previous hidden states, the self-attention module can access features at any position with a constant cost [34]. Most of all, the highly parallel structure of a multi-head attention mechanism is convenient for parallel computation, and gains higher efficiency.

**Figure 6:**
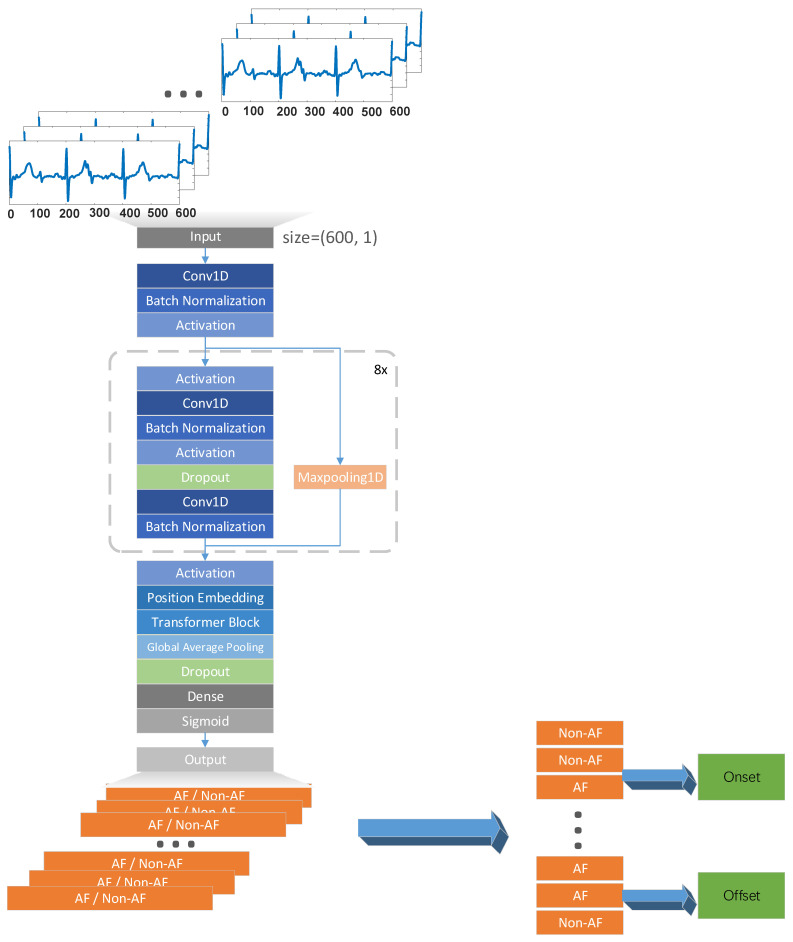
Flowchart of the proposed method. A series of ECG samples were put into the model, and then, the corresponding label sequence could be obtained. The locations of onsets and offsets could be obtained from label sequences. The boundaries between ‘Non-AF’ and ‘AF’ were onsets, while the boundaries between ‘AF’ and ‘Non-AF’ were offsets.

A flowchart of our model can be seen in Figure 6. Here, we used eight residual blocks, followed by the Transformer encoder. We referred to the network proposed by Hannun et al. [35] in 2018. The number of residual blocks was reduced to eight and the Transformer encoder was added. We eliminated decoders because ECG interpretation could be entirely completed by a single encoder, and training the decoder would bring extra time consumption.

Every residual block included two convolution layers, where the deep features of ECG samples were extracted. The kernel size was set to 16. The subsample length was set to 1 for odd residual blocks and 2 for even residual blocks. The number of filters was 32 initially, and it doubled at the fifth residual block. Batch normalization layers followed the two convolution layers. We used ReLU as the activation function. A dropout layer was set to avoid overfitting. Max pooling performed the role of a shortcut to guarantee the same dimensions of input and output. Thus far, the model has grasped the deep features of a 3-beat ECG, but these features describe the ECG in a small range. The mentioned convolution operations can be summarized as an embedding network, concentrating on capturing the latent space representation of ECG signals.

To exploit the potential dependencies between adjacent heartbeats, the outputs of the residual blocks were sent to the position encoding layer to obtain the positional encodings. The dimension of position encoding was 64. We added the position codes to the output of the residual blocks, and fed them into the Transformer encoder. The number of heads was set to 8, and the embedding dimension was 64. The global average pooling (GAP) layer reduced the dimension of the feature map and the parameters of the model, and even prevented overfitting. The data flowed down to the GAP layer, the dropout layer, and sigmoid function successively. The input of the network consisted of the ECG samples of 600 data points. The output was a label sequence representing whether the samples were AF or not. The boundaries between ‘Non-AF’ and ‘AF’ were onsets, while the boundaries between ‘AF’ and ‘Non-AF’ were offsets. We have listed details of the model in Table 2.

Although the feature vectors learned via convolutional computation kept the information in terms of sequence in the spatial domain, less consideration of the relative position weakened the capability of the model when we processed the ECG signals. The Transformer strengthened the ability of position encoding to capture the sequential details, which exactly consolidated the comprehensive performance of model. The encoder also translated the ECG into a more distinguishable form, which benefited the detection of AF. In brief, the combination of convolution and multi-head self-attention provided an insight into not only global features, but also local ones.

## 3. Results

### 3.1. Model Training

The CPSC2021 dataset was used for training. Since the given dataset was unbalanced, we added Gaussian noise to persistent AF recordings for data augmentation. The ECG recordings were segmented by the positions of their R peaks, based on a sliding window, to obtain the ECG samples. The sliding step size was set to one cardiac cycle. The data distribution of the ECG samples in the training set after data augmentation is shown in Table 3.

In total, 80% of the recordings were used for training the model, and 10% for validation in the training phase. The remaining 10% of the recordings were reserved for testing the trained model. Binary cross entropy was used as a loss function, and a Rectified Adam optimizer was chosen for optimization. An early stopping mechanism was set to avoid overfitting. The batch size was set to 128. The model stopped training once the accuracy of the validation set no longer improved within 10 epochs. We trained our models using a NVIDIA GTX 1660 with 6GB memory. The model was built based on TensorFlow and Python.

### 3.2. Metrics

As the proposed method not only discriminated the rhythm of AF from other rhythms, but also predicted the onsets and offsets of the whole recording, we evaluated its performance in the following ways.

Different metrics were used for model evaluation. Common metrics, such as accuracy, sensitivity, specificity, and F1-score, were used to comprehensively measure its performance. FPR (False Positive Rate) was an important indicator to reveal false alarms in the detection of arrhythmias. For the detection of onsets and offsets, we used specific indicators, annotated as Se_onset_ and Se_offset_, to evaluate the results, and Acc_episode_, Se_episode_, Sp_episode_, FPR_episode_, and Mcc_episode_ to describe the detection of episodes. Mcc, in particular, can make the imbalance between TP, FP, TN, and FN clearer. A confusion matrix shows the definitions for classification in Table 4. The mentioned indicators are shown in the following equations.

(4) $$Accuracy=\frac{TP+TN}{TP+TN+FP+FN}$$

(5) $$Sensitivity=\frac{TP}{TP+FN}$$

(6) $$Specificity=\frac{TN}{FP+TN}$$

(7) $$Precision=\frac{TP}{TP+FP}$$

(8) $$F1=\frac{2*Precision*Sensitivity}{Precision+Sensitivity}$$

(9) $$FPR=\frac{FP}{FP+TN}$$

(10) $$Mcc=\frac{TP\cdot TN-FP\cdot FN}{\sqrt{\left(TP+FP)(TP+FN)(TN+FP)(TN+FN\right)}}$$

### 3.3. Model Evaluation and Ablation Experiments

**Figure 7:**
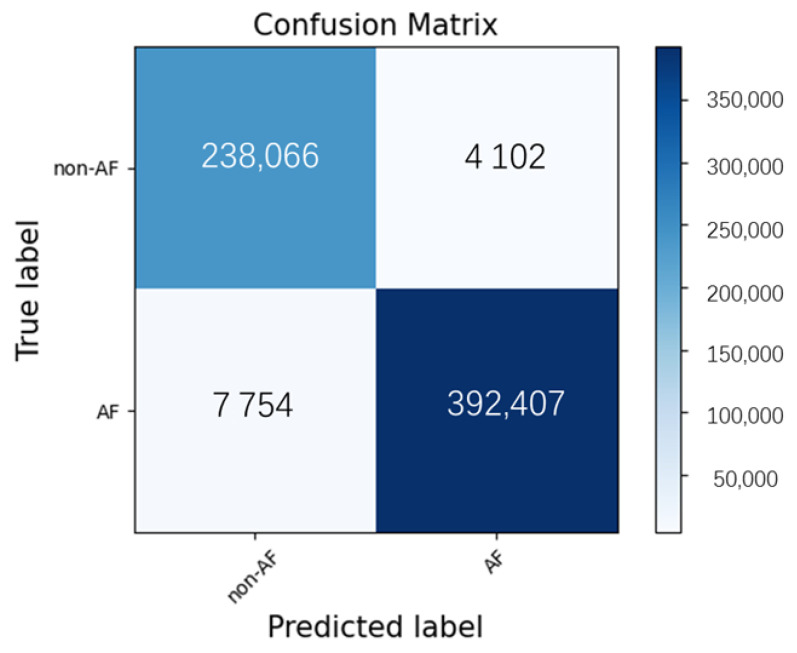
Confusion matrix of binary classification of samples in CPSC2021 test set.

**Figure 8:**
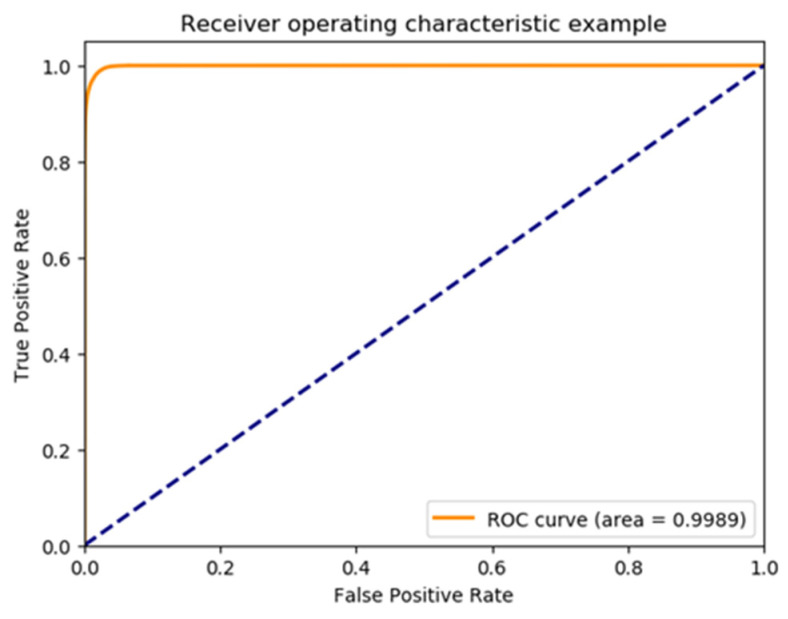
ROC curve and AUC of binary classification for ECG samples in CPSC2021 test set.

During the training phase, the loss value decreased to 0.04. We tested the model on 189 untrained recordings from the CPSC2021 test set. These 189 recordings were composed of 73 AF-excluded, 90 persistent AF, and 26 paroxysmal AF samples. Experiments were carried out to determine the performance of the model in AF discrimination. A total of 642,329 ECG samples were detected. A confusion matrix of the binary classification of rhythm in the CPSC test set is shown in Figure 7. An accuracy of 98.15%, a sensitivity of 98.06%, and a specificity of 98.31% were obtained. The F1-score was 0.9851. ROC and the corresponding AUC of the binary classification for ECG samples from the CPSC test set are shown in Figure 8.

**Figure 9:**
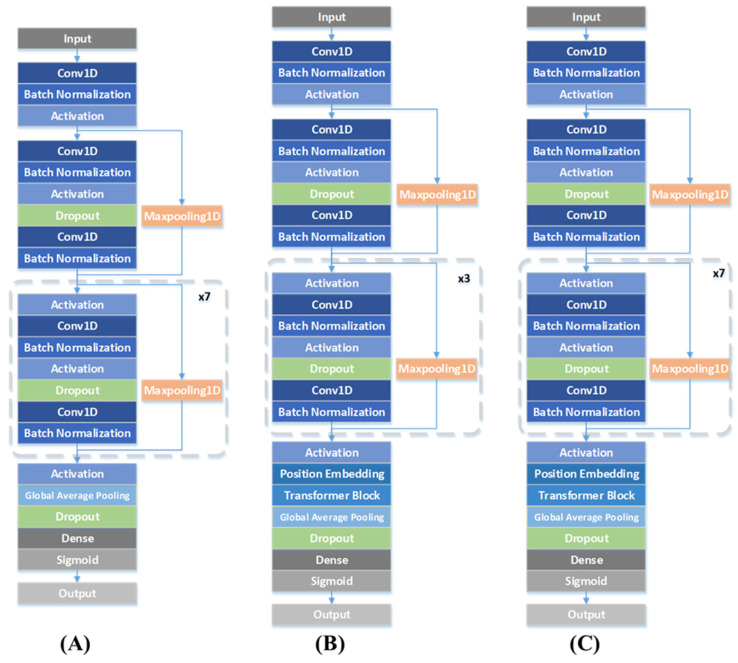
(**A**) Network structure with 8 residual blocks. (**B**) Network structure with 4 residual blocks and Transformer. (**C**) Network structure with 8 residual blocks and Transformer.

We conducted ablation experiments to verify the necessity of residual blocks and the Transformer encoder. We trained two other networks with different structures: (i) eight residual blocks and (ii) four residual blocks and the Transformer (Figure 9). Firstly, we eliminated the Transformer encoder. As shown in Table 5, the values of accuracy, specificity, and F1 were reduced. With almost the same number of parameters, (i) attained a obviously higher FP rate. Since the number of residual blocks was reduced to four in (ii), the accuracy decreased when the parameters were almost halved. However, the testing consumption of the three networks showed a slight difference. In fact, as we were focused on detecting the AF from the short-term ECG, there was no need to sacrifice the accuracy for training consumption; hence, the computational load in the training stage was not our focus.

### 3.4. Performance in AF Rhythm Discrimination and Classification

The trained model was tested on other public AF databases to verify its accuracy using the unseen data, and to test its compatibility and robustness. Here, MITDB, AFDB, LTAF, and Cinc2017 were all used for verification. Except for two unavailable records in AFDB, all the records in these four public databases were used for testing. All the metrics that indicated the model’s performance are shown in Table 6. Both channels were used for evaluation in MITDB, AFDB, and LTAF.

The results on MITDB had an accuracy of 98.32%, a sensitivity of 80.57%, and a specificity of 95.32% for channel 1, and an accuracy of 95.71%, a sensitivity of 65.07%, and a specificity of 97.00% for channel 2. The F1-scores were 0.8539 and 0.7073. The eight recordings with AF were entirely paroxysmal, while the other forty recordings were AF-excluded. The FPRs were 4.67% and 3.00% for channels 1 and 2, respectively. For AFDB, channel 1 obtained an accuracy of 97.91%, a sensitivity of 90.05%, and a specificity of 97.03%. Channel 2 obtained an accuracy of 98.65%, a sensitivity of 90.12%, and a specificity of 97.78%. The FPRs of the two channels were 2.97% and 2.22%, respectively. The F1-scores were 0.8828 and 0.8999 for channels 1 and 2. Next, we performed the same test on LTAF. The values of the indices are summarized in Table 6. For Cinc2017, only one channel was offered, and the corresponding results are also presented in Table 6.

We also merged the results of two channels to obtain a more precise prediction for AF. A voting mechanism was adopted to improve the precision. As for MITDB, an ECG sample was recognized as AF only when the two channels were both predicted as an AF rhythm. If most predicted labels of the samples in a record were inconsistent, one of the channels dominated the final prediction. The model’s performance after merging the labels is presented in Table 7.

### 3.5. Performance in the Detection of AF Onsets, Offsets, and Episodes

Experiments were also carried out to verify the model’s ability to locate AF onset and offset. When a recording was spilt into a series of ECG samples, a series of labels could be obtained (as shown in Figure 4). The locations of AF episodes were provided according to the label sequences. When detected onsets or offsets were within ±3 beats of the annotated position, we considered it correct. Three beats were set as the standard because the whole recording was split to three-beat samples. An ECG sample started from an R-peak and ended at the third one after that, covering three heart beats. As long as one beat in the sample had an AF-like waveform, the sample was classified as AF. Near the onset and offset, a sliding window with an overlapping heartbeat was introduced when splitting the recording into samples, so almost every heartbeat was repeatedly detected three times.

MITDB included eight recordings with AF, and all of them were paroxysmal AF. two recordings in AFDB with persistent AF were excluded, and the remaining twenty-one recordings applied to the test were paroxysmal AF. LTAF included 10 persistent AF recordings, 73 paroxysmal AF recordings, and a recording without AF rhythm. Only paroxysmal AF was reserved when evaluating the performance of boundary detection. The results of onset and offset detection are shown in Table 8. When testing the model’s performance for episode detection, we used all the recordings with AF, whether it was persistent AF or paroxysmal AF. The results of episode detection are shown in Table 9.

### 3.6. Performance on CPSC2021 Hidden Test Data

We took part in the China Physiological Signal Challenge 2021 (CPSC 2021), and our model was examined for hidden test data (see Supplementary Materials). The 4th China Physiological Signal Challenge aimed to encourage the development of algorithms for searching for paroxysmal atrial fibrillation (PAF) events from dynamic ECG recordings. A new dynamic ECG database containing episodes of total or partial AF rhythms, or non-AF rhythms, was constructed to encourage the development of more efficient and robust algorithms for paroxysmal AF event detection [19]. The scoring metric included two steps. The first step was to classify the rhythm types into: non-AF rhythm (N), persistent AF rhythm (AF*_f_*), and paroxysmal AF (AF*_p_*). The second step was to locate the onset and offset for any AF episode prediction. The scoring of step 1 penalized misdiagnoses of the three types of AF. Step 2 allowed deviation within two beats for the prediction of both onset and offset. It was considerate and reasonable to score AF detection with regard to the types of AF and the positions of AF episodes.

**Figure 10:**
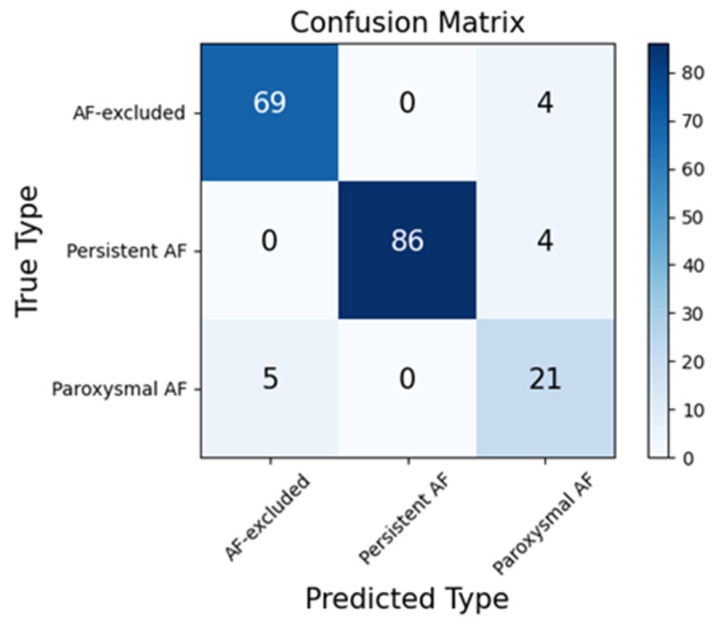
Confusion matrix of three-class classification of recordings in CPSC2021.

When an ECG recording was sent to the model, firstly, every three beats, we obtained a prediction from the aspect of rhythm. According to frequency and duration of AF, the type of recording was confirmed. According to the percentage of the label ‘AF’ and the frequency of consecutive ‘AF’, we classified the recordings into three types: normal, persistent AF, and paroxysmal AF. We added a mechanism to classify the rhythm types: if the percentage of ‘AF’ was less than 0.1, its type would be normal; if the percentage of ‘AF’ was more than 0.9, its type would be persistent AF. The rest would be considered paroxysmal AF. Therefore, recordings were classified into three classes. When the type was AF-excluded or persistent AF, the location was given as null or [1, length of recording]. When the type was paroxysmal AF, we merged the continuous labels and omitted the isolated labels, and then, gave the locations where the AF started and ended. We tested the capability for recording classification on the 189 recordings from CPSC2021, which were not used for training. A confusion matrix of three-class classification in CPSC2021 is shown in Figure 10.

The proposed method was submitted to the CPSC2021 Challenge. Finally, we obtained a score of 1.8754 on test I and 3.5116 on test II, and an average score of 2.6935. We finished in the fourth place in the Challenge, with a narrow gap of 0.0054 points between our score and that of the team in the third place. Our score for the hidden CPSC final test set showed that our proposed method was reliable.

## 4. Discussion

In this study, we developed a deep learning model for the detection of paroxysmal AF. We not only focused on the discrimination between AF rhythm and other rhythms, but also made progress in determining the AF onsets and offsets. The proposed model combined residual blocks and a Transformer encoder. The convolutional layers and the Transformer encoder contributed to feature extraction from different perspectives. Preceding convolutional layers extracted the local characteristic features, while the deeper convolutional layers extracted the features from a higher level. Eight residual blocks were built, and data flowed into two convolutional layers in every residual block. The Transformer encoder was introduced to learn the AF characteristics based on position, which made it possible to construct a connection between data points within a certain distance. Due to the depth of the network, a model was built based on residual blocks in order to avoid gradient vanishing problems. Here, max pooling layers were set as shortcuts to ensure the same dimensions of the module input and output. Through the network, the likelihood of AF could be obtained using the sigmoid function. In this study, ECG signal segments of every three adjacent cardiac cycles were used as input ECG samples for the model. CPSC2021 was used for training and validation, and four public databases, MITDB, AFDB, LTAF, and Cinc2017, were used for testing.

Table 6 summarizes the performance of discrimination between AF rhythm and other rhythms, and we obtained the highest accuracies of 98.32%, 98.65%, and 82.30% on MITDB, AFDB, and LTAF, respectively. The sensitivity values were 80.57%, 90.12%, and 70.35%. The specificity values were 97.78%, 97.00%, and 84.82%. For AFDB and MITDB, the FPR showed values of 2.22% and 3.00%, which indicated that the proposed model could avoid the false alarm problem. The model’s high sensitivity implied a low FN rate, that is, it was less possible for the model to miss an AF rhythm. Hence, the proposed model had a lower probability of failure since high sensitivity and specificity were obtained. We merged the labels from channels 1 and 2, and the performance was obviously improved on AFDB and MITDB. The FPR decreased to 1.44% and 2.00% after we integrated the prediction of the two channels. Accuracy and sensitivity both increased for the two databases.

The model’s performance on MITDB and LTAF decreased, which was mainly due to two reasons. The first reason was that, as shown in Table 6, comparisons were made between channels 1 and 2 in each database. In MITDB and LTAF, channel 1 displayed better performance than channel 2, which was collected from lead V2, V4, or V5. However, the training data were collected from leads I and II, which could explain why better performance was shown when using lead MLII for testing. However, the leads used for data collection in LTAF were undefined. Some ECG recordings had a pattern of ECG waveform that was dissimilar to leads I or II, and we were uncertain about which ECG signals corresponded to which leads. The second reason was that the recordings in MITDB contained different kinds of arrhythmia, that is, 17 types of abnormal beat and 15 kinds of rhythm abnormality. LTAF contained nine types of rhythm. Ectopic beats (PACs/PVCs) are known as AF-masquerading arrhythmias [36]. LTAF had a relatively high proportion of ectopic beats at 3.17% [37]. The ECG recordings used for training were mainly NSR (normal sinus rhythm). The majority of the arrhythmias presented in MITDB and LTAF were not in the training set, which caused the model to confound AF rhythm and other arrhythmias [38,39,40]. A fact that should not be ignored is that there usually exist other arrhythmias if a patient has AF. The model learned the difference between AF and NSR because we used huge amounts of AF and NSR data for training. For records with paced rhythm in MITDB, such as 102, 104, 107, and 217, a high accuracy could be attained only when P waves were obvious. In LTAF and MITDB, the recordings had a considerable number of episodes with atrial bigeminy, ventricular bigeminy, ventricular trigeminy, or ventricular tachycardia. The model’s performance had a negative correlation with the probability of these confused rhythms. Ref. [38] excluded paced beats, bigeminy, and trigeminy from their test set, and their results obviously improved. Ref. [39] also indicated that better performance could be obtained without atrial activity. Ref. [40] concluded that ectopic beats and certain non-AF rhythms caused more FPs in AF detection. Our results on LTAF were consistent with previous research. As a result, the higher incidence rate caused lower precision on LTAF. Bias between sensitivity and specificity indeed existed when the imbalance between positive samples and negative samples was inevitable, which produced an effect on the imbalance between sensitivity and specificity.

**Figure 11:**
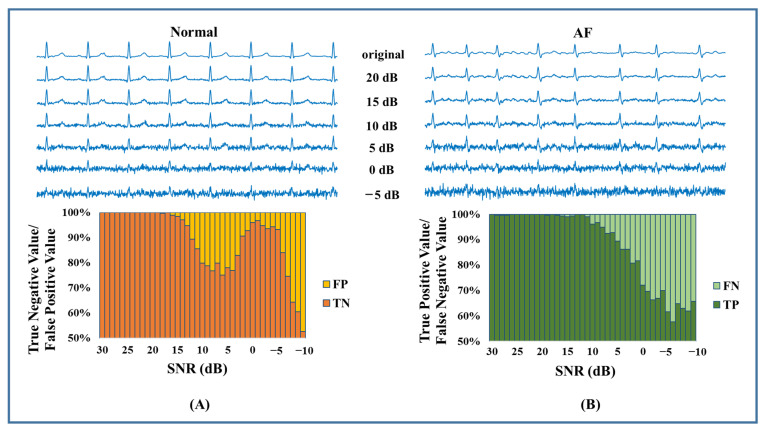
White noise stress test. (**A**) Test result of normal. (**B**) Test result of AF.

**Figure 12:**
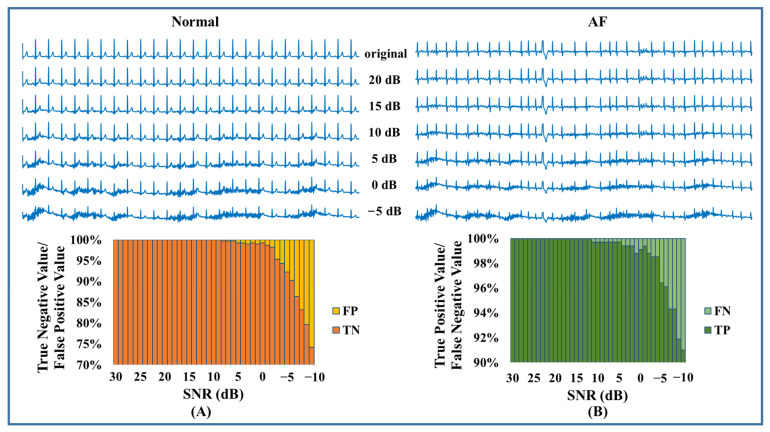
EMG stress test. (**A**) Test result of normal. (**B**) Test result of AF.

**Figure 13:**
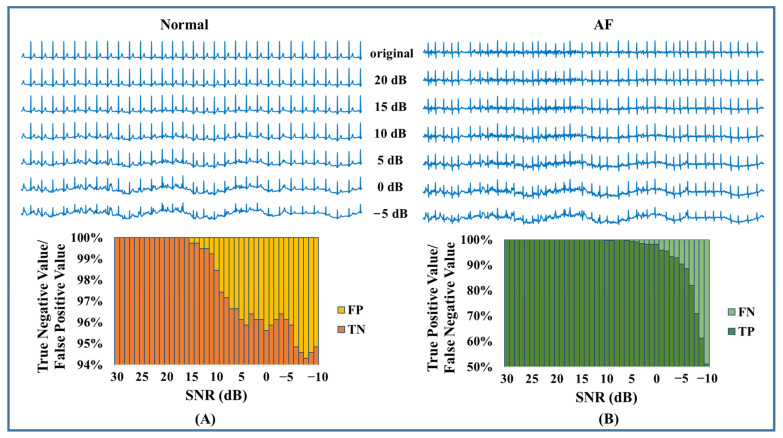
Motion artifact stress test. (**A**) Test result of normal. (**B**) Test result of AF.

For Cinc2017, there were noisy segments mixed into the dataset. Therefore, we used all 8528 recordings to determine whether AF existed. The less accurate detection could be attributed to the noises and artifacts. To further examine the model’s performance in a noisy environment, we conducted a noise stress test by checking the efficiency at different signal-to-noise ratios (SNR). The MIT-BIH Noise Stress Test Database (NSTDB) [41] offered three typical kinds of noise in ambulatory ECG recordings. Here, we only added white noise, EMG, and motion artifacts, because baseline wander can easily be removed. Noise of different levels, ranging from SNR = 30 dB to SNR = −10 dB, was added to the ECG signal (Figure 11, Figure 12 and Figure 13). Since Gaussian white noise was added to augment the training set, our model could tolerate the white noise when the SNR was larger than 15 dB. As shown in Figure 12 and Figure 13, EMG and motion artifacts had a minor effect on the detection of AF, with SNR ≥ 10 dB. The results of the noise stress test above explained why the performance degraded on CinC2017. According to our experience, an ECG signal quality of 10 dB is easy to achieve in practical engineering. It can be concluded that the proposed algorithm is adaptable to practical environments.

The results of boundary and episode detection are presented in Table 8 and Table 9. In AFDB and MITDB, almost the whole boundary was uncovered by the algorithm. The annotated episodes of AF ranged from 2 to 1044 in the recordings from these three databases. The mentioned indicators, such as Acc_episode_, Se_episode_, Sp_episode_, FPR_episode_, and Mcc_episode_, showed the consistency of the predicted label and annotation in MITDB and AFDB.

Here, we present the results of several advanced algorithms for AF detection in Table 10. The model’s performance decreased with data length, since the shorter sample raised the difficulty of AF detection [42]. The proposed method and previous methods had somewhat different objectives. The proposed method aimed to determine the onsets and offsets of AF. However, the aims of the previous methods were to classify an ECG signal into binary categories: Non-AF or AF. Cardiac electrophysiology indicates that AF occurs and terminates suddenly. So, the proposed method needed a short-term sample for high-accuracy detection, i.e., the ECG recordings were split to three-beat samples for the purposes of this study. If the inputs were 5 s or 10 s, brief AF episodes would be missed. This is why we split the ECG recordings into three-beat ECG samples. Previous AF detection methods labeled the input ECG as AF if there existed one beat with AF. A 5 s ECG usually consisted of seven or eight heartbeats, so it would still be labeled as AF if only one beat was AF. Few previous studies have exactly the same objective as the proposed method. We had to list these leading papers to show the competitive performance of our model, and even the detection task was somewhat different. In this paper, we aimed to precisely detect AF in a narrow range and to locate where AF started and ended. Although there is a small gap between the most advanced algorithms, the proposed method is still reasonable. Different authors conduct experiments using different length of samples; hence, we just listed the training and testing consumption for reference. Our model had a similar time cost on computation for one sample when compared with other public results. Although the Transformer had a larger number of parameters than that in previous papers, the model still had competitive efficiency for computational speed. Due to the large amount of training data in CPSC 2021, we spent more time training the model for the detection of AF episodes and its boundaries, while most existing methods only concerned AF detection in longer-term ECGs. We also compared the model’s performance in boundary detection with that of previous paper, which is shown in Table 11. Salinas-Martínez et al. [43] used LTAF for training, so only AFDB and MITDB were compared. They tested their method on two channels of a database. To ensure noticeable results, we made a rule stating that detected onsets and offsets should be within three beats of annotation, and we used several indicators as metrics to determine the model’s performance for boundary and episode detection. However, in Ref. [43], when the ECM images were remapped to the time domain to label each sample, it was obscure because an ECM image included 10 heartbeats. Meanwhile, our model was proven to have the capability for the detection of onsets and offsets, as well as episodes of AF rhythm. The advantage of the proposed method over those used in previous studies is that it can not only detect the boundaries of AF, but also detect episodes of AF.

**Figure 14:**
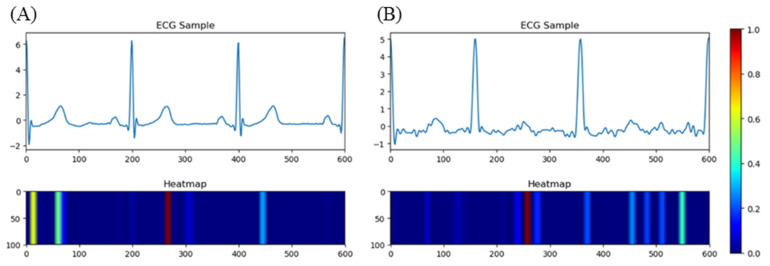
ECG samples and heatmaps for data_39_21 and data_22_14. (**A**) A sample from data_39_21 is NSR. (**B**) A sample from data_22_14 is AF. The red part represents the most important position when the model makes a prediction. The yellow part represents the less important position when the model makes a prediction. In data_39_21, S-wave and T-wave are the most significant regions. In data_22_14, f waves are the most significant region.

**Figure 15:**
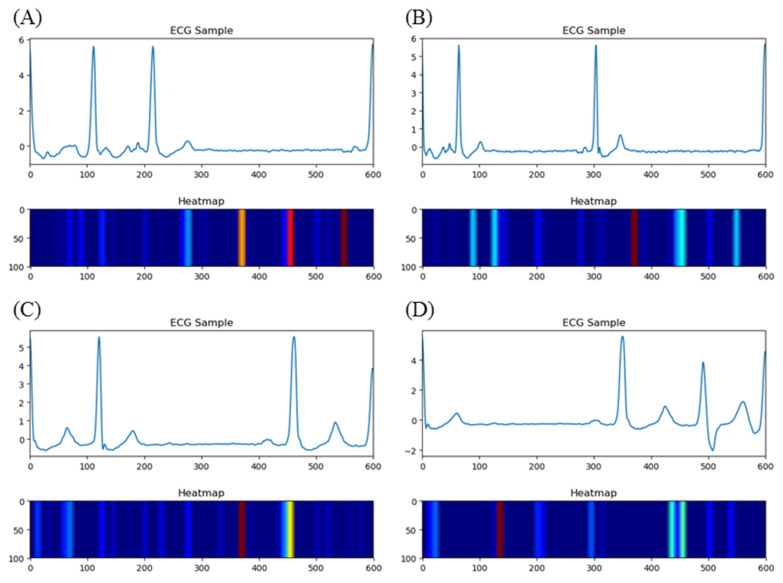
ECG samples and heatmaps for 201 and 202. (**A**) The offset includes two beats with AF and one beat with NSR from 201. (**B**) The offset includes one beat with AF and two beats with NSR from 201. (**C**) The onset includes two beats with NSR and one beat with AF from 202. (**D**) The onset includes one beat with NSR and two beats with AF from 202.

We visualized the importance of every data point in the form of a heatmap and made comparisons between ECG samples with AF rhythm and NSR. The selected ECG samples are shown in Figure 14. We chose data_39_21 and data_22_14 from the CPSC2021 training set. data_39_21 presented no signs of AF rhythm from the first cardiac cycle to the last, so we randomly picked up a sample among them. While data_22_14 showed persistent AF, a sample was also randomly selected. Heatmaps showed that our model focused on the waveform between two R-peaks where signs of AF existed. Here, we found that the T-wave had a close connection with AF detection, which was ignored before. Comparisons were also made between AF onsets and offsets, as shown in Figure 15. In order to determine the model’s performance in the detection of boundaries, records 201 and 202 were used. An offset was offered by record 201. For 201, the first sample included two beats with AF and one beat with NSR, while the second included one beat with AF and two beats with NSR. An onset was also offered by record 202. For 202, the first sample included two beats with NSR and one beat with AF, while the second included one beat with NSR and two beats with AF. The heatmaps imply that the model paid more attention to the adjacent regions of T-waves and the flat waveforms at the positions of P-waves. The heatmaps made the prediction conducted via deep learning strategies transparent and explainable, and the features learned by the model made sense in the physiological field. Advances in the detection of AF will likely yield inexpensive and practical options for AF burden assessment in the near future. Early AF screening in high-risk and elderly populations can lead to comprehensive AF diagnosis and management. AF detection with fewer leads has practical applications in engineering, such as in wearable ECG monitor where only fewer leads are available.

## 5. Limitations

The training data we used were collected from lead I and lead II, and most of them included little noise, which could probably cause false prediction. The rhythms shown in the training data were mainly NSR and AF. The proposed model was proven to achieve good performance when the above conditions were satisfied. When data were from other leads or had complicated rhythms, the performance of the proposed model could be limited. In the future, we intend to absorb all 12 leads for further study. The model’s generalizability could be significantly improved if all of the 12-lead ECG data with various arrhythmias were added to the training set. The model’s performance on the CPSC hidden test set suggested that it has the ability to discriminate AF from NSR under the condition of adequate signal quality, while its performance on Cinc2017 implied an improvement in noisy environments.

## 6. Conclusions

In this study, we proposed a deep learning model for AF rhythm detection, both for onsets and offsets. The model was built based on eight residual blocks and a Transformer encoder. We trained the model on CPSC2021, and tested it on four public datasets. The best performance for AF rhythm testing attained an accuracy of 98.67%, a sensitivity of 87.69%, and a specificity of 98.56%, respectively. The performance for onset and offset detection obtained a sensitivity of 95.90% and 87.70%. The algorithm with a low FPR of 0.46% was able to reduce troubling false alarms. Finally, we visualized the features of the heatmap and illustrated its interpretability. The proposed model can be relied upon for AF detection, which is commonly used in dynamic ECG devices.

## Figures and Tables

**Table 1:** Statistics of the used databases.

	CPSC 2021	AFDB	LTAF	MITDB	CinC 2017
Non-AF Records	732	0	1	40	7757
Persistent AF Records	475	2	8	0	/
Paroxysmal AF Records	229	21	73	8	/
Average Duration of Record	0.3 h	10 h	24~25 h	0.5 h	/
Average Beats in a Record	1356	49,068	107,810	2346	/
Duration of Record	8 s~6 h	10 h	24~25 h	0.5 h	9~60 s
Total Beats	2,146,915	1,128,561	9,055,636	112,646	/
Subjects	49 AF (23 PAF) 56 non-AF	/	/	47	/
Episodes with Paroxysmal AF	677 (≥5 beats)	285	7358	122	/
Beats with AF	770,396	520,394	3,118,292	13,259	/
Number of Records	1436	23 (2 unavailable)	84	48	8528
Sample Rate (Hz)	200	250	128	360	300
Label (Types of Rhythm)	AF/AFL/normal	AFIB/AFL/ J/Others	9 types	15 types	Normal/ AF/ Others/ noise
Lead	I, II	/	/	MLII, V1, V2, V4, V5	/
Sources	12-lead Holter or 3-lead wearable ECG monitoring devices	ambulatory ECG recorder	/	24 h ambulatory ECG	AliveCor device

**Table 2:** Details of the model.

Layer	Output Shape	Parameters
Inputs	(600, 1)	0
Conv1D	(600, 32)	544
Batch Normalization	(600, 32)	128
ReLU	(600, 32)	0
Conv1D in Block 1–Block 4	(none, 32)	16,416
Batch Normalization in Block 1–Block 4	(none, 32)	128
Conv1D in Block 5–Block 8	(none, 64)	65,600
Batch Normalization in Block 5–Block 8	(none, 64)	256
Position Encoding	(38, 64)	3200
Transformer Encoder	(38, 64)	21,088
Global Average Pooling1D	(none, 64)	0
Dense	(none, 32)	2080
Dropout	(none, 32)	0
Dense	(none, 1)	33
Outputs	(none, 1)	0
Total Parameters		653,505
Trainable Parameters		651,905

**Table 3:** Data distribution of ECG samples in training set after data augmentation.

	Non-AF	AF	Total of Samples
Normal	1,258,848	0	1,258,848
Persistent AF	0	675,477	675,477
Paroxysmal AF	117,442	94,919	212,590
Total Samples	1,375,558	769,921	2,145,479
Total (After Augmentation)	1,375,558	1,445,873	2,821,431

**Table 4:** Confusion matrix for classification.

	Prediction	
Real Label	Positive	Negative
Positive	TP (True Positive)	FN (False Negative)
Negative	FP (False Positive)	TN (True Negative)

**Table 5:** Comparisons of performance of different models on CPSC2021.

Model	Accuracy (%)	Sensitivity (%)	Specificity (%)	FPR (%)	F1- Score	Params	Training Consumption	Testing Consumption
8 Res Blocks	97.83	98.80	97.08	2.92	0.9753	627,617	31.3 h	45 min
4 Res Blocks + Transformer	97.98	98.84	97.32	2.68	0.9771	323,073	29.8 h	40 min
8 Res Blocks + Transformer	98.15	98.06	98.31	1.59	0.9851	651,905	33.6 h	45 min

**Table 6:** Performance on public databases.

Database		Records	Accuracy (%)	Sensitivity (%)	Specificity (%)	FPR (%)	F1-Score
CPSC2021		189	98.15	98.06	98.31	1.59	0.9851
MITDB	CH1	48	98.32	80.57	95.32	4.67	0.8539
	CH2	48	95.71	65.07	97.00	3.00	0.7073
LTAF	CH1	84	82.30	70.35	84.82	15.18	0.7401
	CH2	84	76.67	64.88	71.33	28.66	0.6292
AFDB	CH1	23	97.91	90.05	97.03	2.97	0.8828
	CH2	23	98.65	90.12	97.78	2.22	0.8999
CinC2017		8528	89.29	79.84	91.09	8.81	0.7048

**Table 7:** Performance after merging the labels.

Database	Records	Accuracy (%)	Sensitivity (%)	Specificity (%)	FPR (%)	F1-Score
MITDB	48	96.34	80.57	97.99	2.00	0.8539
LTAF	84	86.16	65.71	83.09	16.91	0.7401
AFDB	23	98.67	87.69	98.56	1.44	0.9008

**Table 8:** Results of onset and offset detection.

Database	MITDB	AFDB	LTAF
Episodes	122	285	7358
Detected Onset	117	260	4709
Detected Offset	107	227	4424
Se_Onset_ (%)	95.90	91.23	64.00
Se_Offset_ (%)	87.70	79.65	60.13

**Table 9:** Results of episode detection.

Database	AFDB		MITDB		LTAF	
	CH1	CH2	CH1	CH2	CH1	CH2
Episodes	122	122	285	285	7358	7358
Acc_episode_ (%)	93.34	93.93	97.69	96.62	77.41	68.02
Se_episode_ (%)	83.42	83.12	80.00	65.00	69.07	54.87
FPR_episode_(%)	1.55	0.46	4.17	3.47	18.12	13.43
Mcc_episode_	86.35	76.04	89.75	63.47	57.97	58.35

**Table 10:** Results of several advanced algorithms for AF detection.

	ECG Length	Database	Accuracy (%)	Sensitivity (%)	Specificity (%)	F1-Score	Training Consumption	Testing Consumption (Every Sample)
[16]	10 s	CPSC 2018	99.35	99.44	99.19	0.9906	19.7 min	2.7 ms
[43]	10 beats	AFDB	87.88	84.56	90.84	0.8686	/	/
[14]	5 s	AFDB	98.81	99.08	98.54	/	/	/
[44]	5 s	AFDB	98.51	98.14	98.76	/	122 s/epoch	0.6 ms
[13]	5 s	AFDB	98.29	98.34	97.87	/	40 min	/
[45]	1 beat	AFDB	/	96.68	98.4	0.9705	44 s/epoch	/
Our method	3 beats	AFDB	98.69	87.69	98.56	0.9008	/	1.1 ms
		AFDB (CH1)	97.91	90.05	97.03	0.8828	/	0.52 ms
		AFDB (CH2)	98.65	90.12	97.78	0.8999	/	0.52 ms

**Table 11:** Comparisons for boundary and episode detection.

Database	Metrics	Our Method	Salinas-Martínez et al. [24]
AFDB	Se_Dur_ (%)	90.05–90.12	75.95–86.71
	PPV_dur_ (%)	85.21–88.43	89.85–93.40
	Se_episode_ (%)	83.18–83.42	96.73–97.45
	PPV_episode_ (%)	94.13–95.23	61.10–80.15
	FPR_episode_ (%)	0.46–1.55	-
	Se_onset_ (%)	91.23	-
	Se_offset_ (%)	79.65	-
MITDB	Se_dur_ (%)	65.07–80.57	85.26–95.32
	PPV_dur_ (%)	26.89–29.46	31.05–31.50
	Se_episode_ (%)	65.00–80.00	90.65–98.13
	PPV_episode_ (%)	30.80–57.86	8.32–12.99
	FPR_episode_ (%)	3.47–4.17	-
	Se_onset_ (%)	95.90	-
	Se_offset_ (%)	87.70	-

## Data Availability

Public datasets are listed as following: Wang, X., Ma, C., Zhang, X., Gao, H., Clifford, G. D., & Liu, C. (2021). Paroxysmal Atrial Fibrillation Events Detection from Dynamic ECG Recordings: The 4th China Physiological Signal Challenge 2021 (version 1.0.0). PhysioNet. https://doi.org/10.13026/ksya-qw89. Moody, G.B.; Mark, R.G. The impact of the MIT-BIH Arrhythmia Database. IEEE Eng. Med. Biol. 2001, 20, 45–50. Moody GB, Mark RG. The impact of the MIT-BIH Arrhythmia Database. IEEE Eng in Med and Biol 20(3):45–50 (May–June 2001). (PMID: 11446209). Petrutiu S, Sahakian AV, Swiryn S. Abrupt changes in fibrillatory wave characteristics at the termination of paroxysmal atrial fibrillation in humans. Europace 9:466–470 (2007). Moody GB, Mark RG. A new method for detecting atrial fibrillation using R-R intervals. Computers in Cardiology. 10:227–230 (1983). Clifford GD, Liu C, Moody B, Li-wei HL, Silva I, Li Q, Johnson AE, Mark RG. AF classification from a short single lead ECG recording: The PhysioNet/computing in cardiology challenge 2017. In 2017 Computing in Cardiology (CinC) 2017 Sep 24 (pp. 1–4). IEEE. https://doi.org/10.22489/CinC.2017.065-469. Moody GB, Muldrow WE, Mark RG. A noise stress test for arrhythmia detectors. Computers in Cardiology 1984; 11:381–384.

## Supplementary Materials

Our code for the CPSC2021 Challenge is available on GitHub: https://github.com/hhhh59/cpsc2021-python-entry (accessed on 9 May 2023).
